# DMH1, a Small Molecule Inhibitor of BMP Type I Receptors, Suppresses Growth and Invasion of Lung Cancer

**DOI:** 10.1371/journal.pone.0090748

**Published:** 2014-03-06

**Authors:** Jijun Hao, Rachel Lee, Andy Chang, Jeffery Fan, Chantelle Labib, Cyrus Parsa, Robert Orlando, Bradley Andresen, Ying Huang

**Affiliations:** 1 College of Veterinary Medicine, Western University of Health Sciences, Pomona, California, United States of America; 2 College of Pharmacy, Western University of Health Sciences, Pomona, California, United States of America; 3 College of Biomedical Sciences, Western University of Health Sciences, Pomona, California, United States of America; 4 Department of Clinical Sciences, College of Osteopathic Medicine, Western University of Health Sciences, Pomona, California, United States of America; Rush University Medical Center, United States of America

## Abstract

The bone morphogenetic protein (BMP) signaling cascade is aberrantly activated in human non-small cell lung cancer (NSCLC) but not in normal lung epithelial cells, suggesting that blocking BMP signaling may be an effective therapeutic approach for lung cancer. Previous studies demonstrated that some BMP antagonists, which bind to extracellular BMP ligands and prevent their association with BMP receptors, dramatically reduced lung tumor growth. However, clinical application of protein-based BMP antagonists is limited by short half-lives, poor intra-tumor delivery as well as resistance caused by potential gain-of-function mutations in the downstream of the BMP pathway. Small molecule BMP inhibitors which target the intracellular BMP cascades would be ideal for anticancer drug development. In a zebrafish embryo-based structure and activity study, we previously identified a group of highly selective small molecule inhibitors specifically antagonizing the intracellular kinase domain of BMP type I receptors. In the present study, we demonstrated that DMH1, one of such inhibitors, potently reduced lung cell proliferation, promoted cell death, and decreased cell migration and invasion in NSCLC cells by blocking BMP signaling, as indicated by suppression of Smad 1/5/8 phosphorylation and gene expression of Id1, Id2 and Id3. Additionally, DMH1 treatment significantly reduced the tumor growth in human lung cancer xenograft model. In conclusion, our study indicates that small molecule inhibitors of BMP type I receptors may offer a promising novel strategy for lung cancer treatment.

## Introduction

Lung cancer is one of the most common types of cancer and the leading cause of cancer deaths. About 228,190 cases of lung cancer are expected to be newly diagnosed in 2013, accounting for ∼27% of all cancer deaths annually in the US [1]. The major type of lung cancer, non-small cell lung cancer (NSCLC), comprises approximately 85% of all diagnosed lung cancers. Despite improvements in the diagnosis and chemotherapy, 5-year survival rate for patients with NSCLC is still very low. Recently, great progresses have been made in the understanding of the molecular mechanisms driving lung cancer development, which resulted in a few targeted therapies [2]. However, the patients who respond initially invariably relapse. There is a need to identify novel targets for NSCLC.

Bone morphogenetic proteins (BMPs) are members of the TGF-β superfamily and their biological activity is mediated through the formation of heterodimeric complexes of the BMP type I and type II serine/threonine kinases receptors. After the ligand binding, the BMP type I receptors are phosphorylated by the constitutively active type II receptors, leading to phosphorylation of the intracellular Smad 1/5/8 proteins, which then form a complex with Smad4 and translocate into the nucleus to regulate transcriptional response [3], [4]. Over 20 BMP ligands have been identified to date [5]. Overexpression of BMP-2 has been associated with ∼98% of NSCLC and other types of malignancy [6], [7]. In addition, forced expression of BMP-2 in NSCLC cell lines significantly enhanced tumor growth in a mouse model of lung cancer following tail intravenous injection of tumor cells [8]. Conversely, the BMP antagonist Noggin and the extracellular pseudoreceptor spp24 (secreted phosphoprotein 24 kD) dramatically reduced lung tumor growth in subcutaneous xenograft mouse models [9], [10], suggesting that inhibition of the BMP signaling may be an effective therapy for lung cancer. However, the protein-based BMP antagonists or pseudoreceptor spp24 mainly interfere the binding of extracellular BMP ligands to their receptors. Their clinical application could be limited by potential gain-of-function mutations in the downstream members of the BMP signaling cascade or short half-lives and poor delivery to tumors which are common problems associated with protein-based therapy.

**Figure 1:**
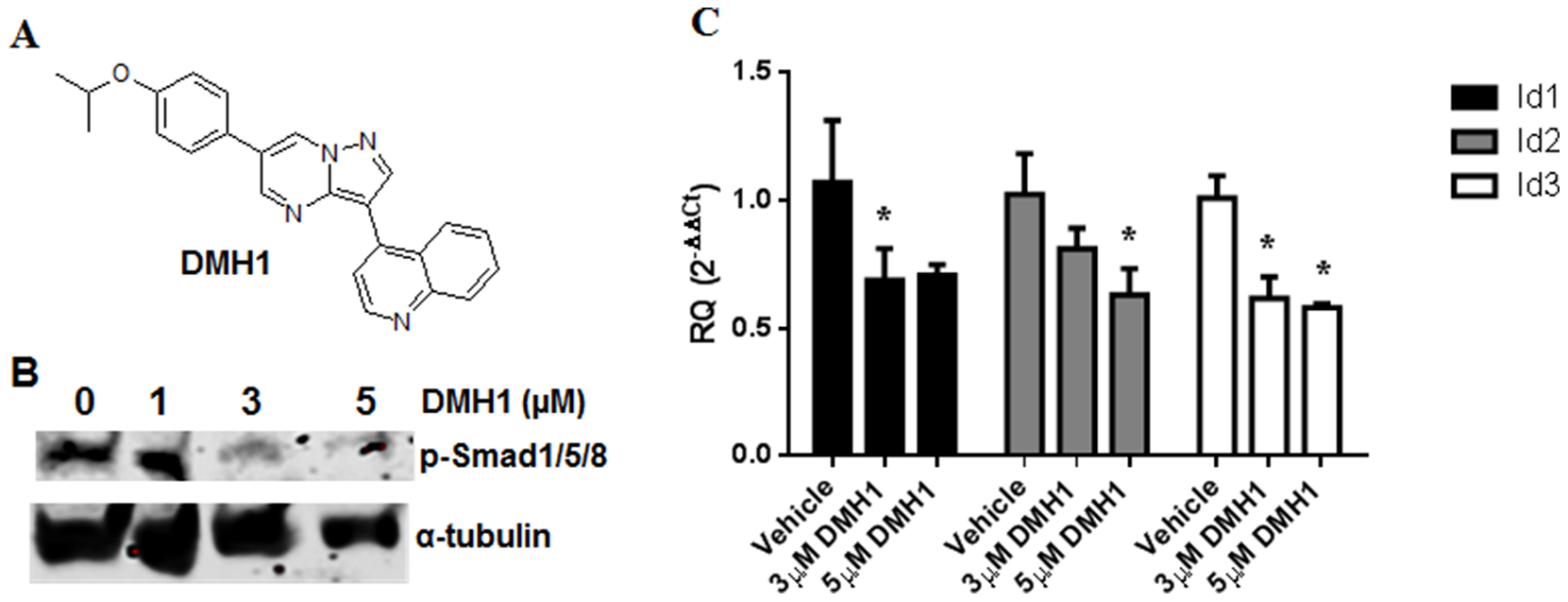
DMH1 inhibits basal Smad phosphorylation and transcription of Id genes in NSCLC cells. Chemical structure of DMH1; (B) Western blotting for phosphorylated Smad1/5/8 in A549 cells treated with DMH1 at various concentrations (1, 3, and 5 µM); (C) QPCR results of Id1, 2 and 3 in A549 cells treated with DMH1 and vehicle DMSO. *: *p*-value is <0.05.

In an *in vivo* structure-activity relationship study based on a zebrafish embryonic development model, we previously identified a group of highly selective small molecular BMP inhibitors including DMH1 and DMH2, which specifically block BMP signaling by targeting the intracellular kinase domain of BMP type I receptors [11] (the structure of DMH1 is shown in **Figure 1A**). A very recent study reported that DMH2, one of our BMP inhibitors, effectively decreased growth and induced cell death of NSCLC cells *in vitro* [12]. However, *in vivo* study of small molecular BMP inhibitors on NSCLC tumor growth has not been reported. As DMH1 displays a better selectivity for BMP type I receptors than DMH2 [11], in the present study we investigated the effects of DMH1 on cell proliferation, migration and invasion of the NSCLC cell lines *in vitro* as well as on the xenograft lung tumor growth in mice. Our study demonstrated that DMH1 was able to significantly reduce NSCLC cell growth, migration and invasion, and attenuate xenograft lung tumor growth *in vivo*.

## Materials and Methods

### Cell culture and reagents

A549 and H460 cells were purchased from American Type Culture Collection (ATCC, Manassas, VA), cultured in RPMI 1640 supplemented with 10% FBS (Gibco) and 1% penicillin-streptomycin (Cellgro) in an atmosphere of 5% CO_2_ at 37°C.

### Western blotting

Cells were lysed in RIPA cell lysis buffer (Santa Cruz, Santa Cruz, CA) supplemented with protease inhibitor cocktail (Santa Cruz) and phosphatase inhibitor cocktail 2 (Santa Cruz). Protein concentrations were determined by using BCA protein kit (Thermo Fisher), and cell lysate was isolated in SDS-PAGE and transferred onto PVDF membrane. Alpha-tubulin and p-Smad1/5/8 were detected by Odyssey system (Li-Cor bioscience) following incubation with the appropriate primary and secondary antibodies. Primary antibodies used were rabbit anti-p-Smad1/5/8 (Cell Signaling Tech, 1∶1000 dilution) and mouse anti-alpha-tubulin (Cell Signaling Tech, 1∶1000 dilution). The secondary antibodies used include IRDye 680-conjugated goat anti-rabbit IgG (Li-Cor Bioscience, 1∶5000 dilution) and IRDye 800CW-conjugated goat anti-mouse IgG (Li-Cor Bioscience, 1∶5000 dilution).

### Cell scratch-wound Assay

A549 and H460 cells were seeded in 35 mm dishes to create a confluent monolayer. The dishes were allowed to incubate overnight in order to allow the cells to attach to the bottom of the dish. On the following day, wounds were created by a straight scratch from a pipette tip in the center of the culture. The cells were then treated with DMSO or DMH1 at 1 µM and 3 µM concentrations. Photographs were taken when wounds were created and after 24 hour's incubation using phase-contrast microscopy, and gap distances were quantitatively evaluated using software ImageJ (NIH). The gap distances after 24 h incubation were normalized with the gap distance at 0 hr as the migration rates.

### Cell Proliferation Assay

About 10,000 A549 cells per well were seeded in 96-well plates and incubated for overnight. The culture medium was then changed to fresh medium containing DMSO or DMH1 at various concentrations. The cells were then incubated for 48 hours and 96 hours before treatment termination by replacing the medium with 100 μL of 10% trichloroacetic acid (Sigma) in 1× PBS, followed by incubation at 4°C for at least 1 hour. Subsequently, the plates were washed with water and air dried. The plates were stained with 50 μL 0.4% sulphorhodamine (SRB, Sigma) assay in 1% acetic acid for 30 minutes at room temperature. Unbound dye was washed off with 1% acetic acid. After air drying and solubilization of the protein-bound dye in 10 mM Tris solution, absorbance was read in a microplate reader at 565 nm.

### Quantitative real-time PCR

A549 and H460 cells seeded in 6-well plates at a density of 3×10^5^ cells per well were treated with DMSO or DMH1 for 24 hours. Total RNA was extracted using TRIzol Reagent (Invitrogen) following manufacturer's protocol. cDNA was synthesized using the High Capacity cDNA Reverse Transcription Kit (Applied Biosystems). Real-time PCR was carried out using the Power SYBR Green PCR master mix (Applied Biosystems) and with the Applied Biosystems 7300 PCR System. All amplification controls and samples were performed in triplicate. The primer sequences are available upon request.

### Modified Boyden chamber assay

Cell invasion was measured using a 24-Multiwell Insert System (8 µM membrane, BD Biosciences) according to the manufacture instruction. The cell culture inserts were coated with matrigel (BD Biosciences). A549 cells were seeded at a concentration of 3×105 cells/chamber, After 24-h incubation with or without DMH1 (3 μM), cells that had not moved to the lower wells were removed from the upper face of the filters using cotton swabs, and the cells that invaded through the matrigel-coated-inserts were counted. Mean values for three randomly selected fields were obtained for each well. Experiments were performed in duplicate. Mean values for three random fields were obtained for each well.

### Xenograft lung tumor growth

This study was carried out in strict accordance with the recommendations in the Guide for the Care and Use of Laboratory Animals of the National Institutes of Health. The animal experimental protocol was approved by the Western University of Health Sciences Institutional Animal Care and Use Committees (IACUC). All efforts were made to minimize animal suffering. Sub-confluent A549 cells were trypsinized and then suspended in serum free RPMI 1640 medium. The cell suspension (1×10^6^ cells in 100 µl medium for each injection) was injected subcutaneously into both the right and left flanks of eight-week old NOD SCID mice (Taconic, Hudson, NY) (n = 5 for each group). Mice were given Intraperitoneal (i.p.) injection of the vehicle (12.5% 2-hydroxypropyl-β-cyclodextrin) or 5 mg/kg DMH1 every other day. The tumor sizes were measured with a vernier caliper from the sixth day to the fourth week after tumor implantation. The tumor volume (V) was calculated according to the formulation: Volume  =  (width)∧2× length/2. The tumor tissues were dissected at the end of study, and were sectioned and stained with H & E, and for immunohistochemical analysis.

### Tumor sample analysis

Lung tumor tissues from both the vehicle and DMH1 treated mice were excised and fixed immediately in formalin and embedded in paraffin blocks. The embedded tumors were cut into 3 micron thick sections and stained with H&E to determine morphology. Cell proliferation in the tumors was performed at Pathology Inc. Laboratories (Torrance, California) detected by immunostaining with an antibody to Ki67 (Dako MIB-1 Clone, Carpinteria, California), used at 1∶50 on Leica Bond III IHC staining system. The stained sections were visualized and photographed with a Nikon microscope image capture system (Nikon DS-Ri1) and public domain software (NIH Image v1.60).

### Statistical analysis

Data are expressed as mean ± standard error. The data was analyzed using NCSS 2007 (Kaysville, UT) via t-test for comparison of two means, one way ANOVA with Fisher's LSD post-hoc test for comparison of multiple means, and repeated measures ANOVA with Tukey-Kramer post-hoc test for the *in vivo* xenograph studies. The data was graphed and curve fitting was analyzed with GraphPad Prism version 6 (La Jolla, CA). For all statistical analysis, means were indicated to be statistically different when *p*<0.05.

## Results

### DMH1 blocks BMP signaling in NSCLC cells

We first examined whether DMH1 can block BMP signaling in NSCLC cells using Western blotting and quantitative real-time PCR (qPCR). NSCLC A549 cells were treated with the vehicle (DMSO) or DMH1 at 1, 3, and 5 μM for 24 hours, respectively. Western blotting analysis indicated that A549 cells displayed relatively high basal phospho-Smad 1/5/8 expression, and DMH1 treatment effectively blocked Smad 1/5/8 phosphorylation in a dose dependent manner (**Figure 1B**). Since the Inhibitors of DNA binding/differentiation (Id) family members are direct mediators of the BMP signaling [13], and they are involved in invasion, proliferation and metastasis of cancer cells [14], we measured the gene expression of Id1, Id2 and Id3 in A549 by qPCR. After 24 hours treatment, 3 and 5 μM DMH1 robustly decreased the expression of all the three genes (**Figure 1C**). Taken together, DMH1 can effectively block BMP signaling in NSCLC cells.

### DMH1 decreases NSCLC cell migration and invasion

**Figure 2:**
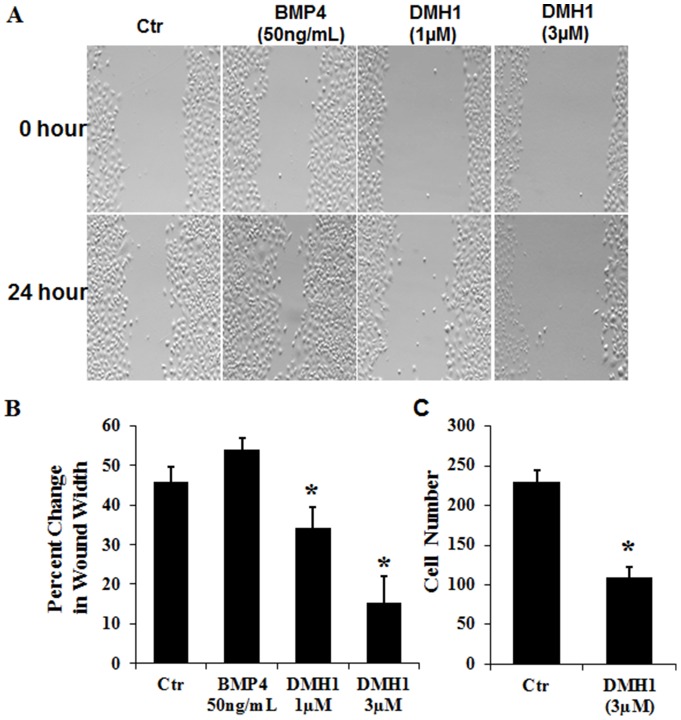
DMH1 decreases NSCLC cell migration and invasion. Representative images from scratch-wound assays performed in the cultured A549 cells treated with DMSO, BMP4 and DMH1 (1 µM and 3 µM) for 24 hours. (B) Cell migrations of A549 cells were quantified by the gap distances after 24 hour treatment normalized with the initial gap distances. (C) Cell migrations of H460 cells were quantified in a similar way. (D) The effect of DMH1 (3 µM) on A549 cell invasion was determined using modified Boyden chamber assay in a 24-Multiwell Insert System (8 µM membrane, BD Biosciences) coated with matrigel. *: *p*-value is <0.05.

Since cell migration and invasion are known to play an important role in the progression of cancer metastasis, we examined the effects of DMH1 on NSCLC cell migration and invasion *in vitro*. We used the scratch-wound assay to determine NSCLC cell migration by creating wound gaps in the cultured A549 cells [15]. Cells were then treated with DMSO, one of the BMP ligands BMP4 or DMH1 for 24 hours respectively, and the gap distances were then normalized with the initially measured distances. BMP4 was used because it has shared function with BMP2 but with higher potency. As shown in **Figure 2A and 2B**, BMP4 notably expedited cell migration whereas DMH1 dramatically slowed down migration in a dose-dependent manner. Similar effects of BMP4 and DMH1 on cell migration were also observed in another NSCLC cell line H460 (**Figure 2C**). In addition, we examined the effect of DMH1 on cell invasion by using modified Boyden chamber assay. A549 cells were seeded on matrigel-coated chambers, followed by 24-h incubation with or without DMH1. 3 µM DMH1 dramatically reduced A549 cell invasion through matrigel-coated membranes by about 52% in comparison with the vehicle controls (**Figure 2D**).

### DMH1 reduces NSCLC cell proliferation and induces cell death

**Figure 3:**
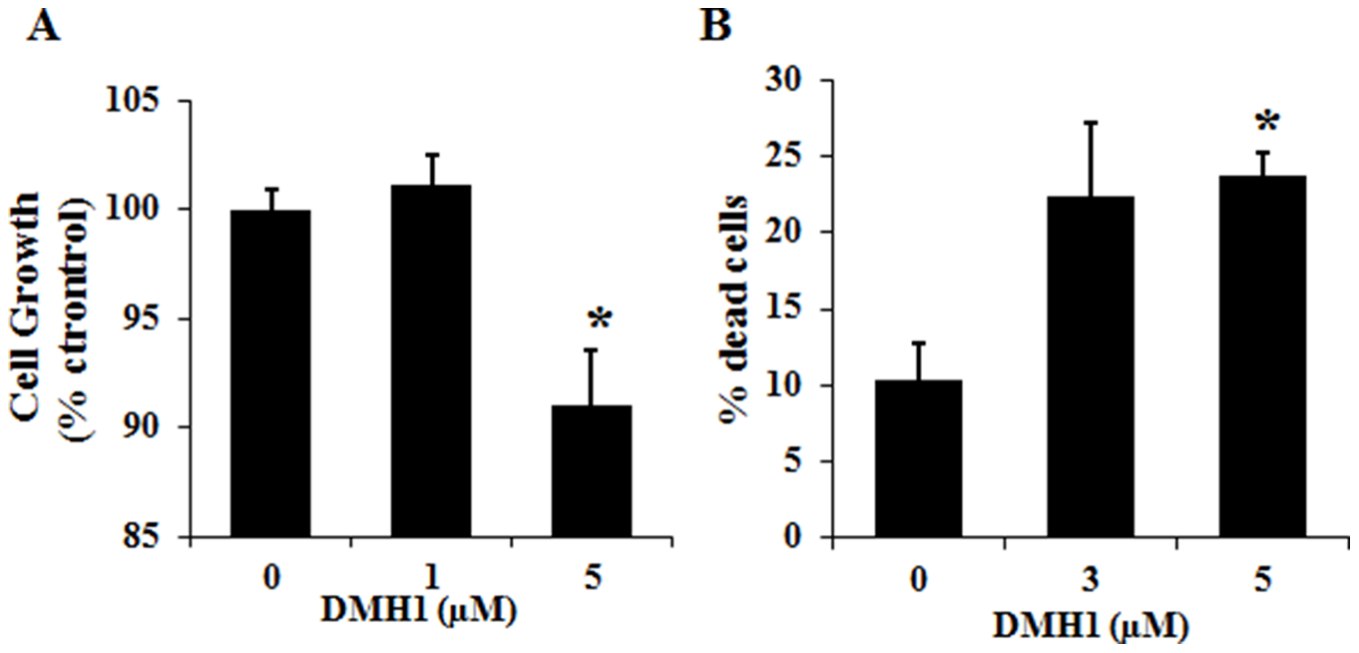
DMH1 reduces A549 cell proliferation and induces cell death. A549 cells were treated with DMH1 (3 and 5 µM) or DMSO for 48 hours and cell proliferation was determined by the sulforhodamine B (SRB) assay. (B) A549 cells were treated with DMH1 or DMSO for 72 hours, and cells were harvested for cell death assay using Trypan Blue staining. *: *p*-value is <0.05.

We next examined the effects of DMH1 on NSCLC cell proliferation and survival. A549 cells were treated with DMH1 and the vehicle DMSO for 48 hours, and cell proliferation was determined by the sulforhodamine B (SRB) assay. The result showed that 5 µM DMH1 led to about 10% reduction in cell growth after two day treatment, suggesting that inhibition of BMP signaling by DMH1 significantly reduces lung cancer cell proliferation *in vitro* (**Figure 3A**). In addition, we examined the effect of DMH1 on A549 cell survival as well. A549 cells were treated with DMH1 or vehicle DMSO for 72 hours, and floating and adherent cells were harvested and stained with Trypan Blue to determine the number of dead and dying cells. In contrast to the vehicle treatment, DMH1 significantly increased the percentage of dead cells at 5 µM concentrations, suggesting that antagonizing BMP signaling by DMH1 significantly increases lung cancer cell death (**Figure 3B**). Thus, DMH1 treatment can dramatically reduce NSCLC cell proliferation and induces cell death *in vitro.*

### DMH1 attenuates xenograft lung tumor growth in mice

**Figure 4:**
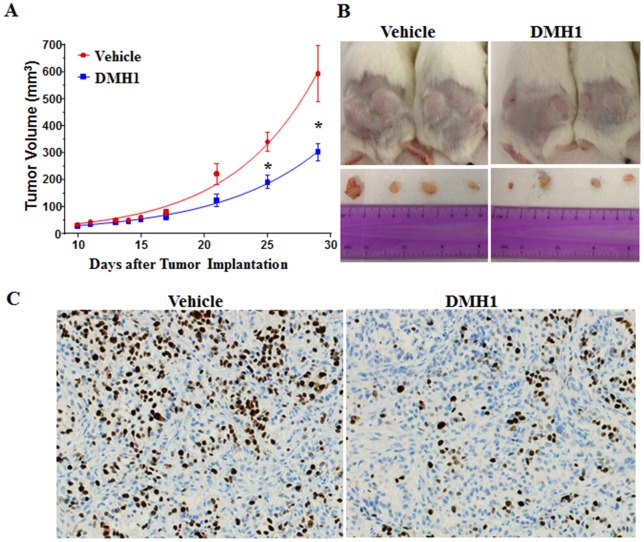
DMH1 attenuates xenograft lung tumor growth in mice. The A549 cells were subcutaneously implanted into the SCID mice followed by intraperitoneal injection of the vehicle (12.5% 2-hydroxypropyl-β-cyclodextrin) or 5 mg/kg DMH1 every other day for 4 weeks. Tumor volumes were measured from the sixth day to four weeks after injection. (B) Representative tumor tissues from mice treated with the vehicle control and DMH1 are compared. (C) Tumor tissues were stained for human specific Ki67 proliferation marker. *: *p*-value is <0.05.

We next examined the effect of DMH1 on lung tumor cell growth *in vivo*. The A549 cells were subcutaneously inoculated in the two sides of lower rear flanks of Severe combined immunodeficiency (SCID) mice. Intraperitoneal (i.p.) injections of vehicle (12.5% 2-hydroxypropyl-β-cyclodextrin, n = 5) or 5 mg/kg DMH1 (n = 5) were initiated on the same day of tumor cell implantation and were performed every other day for 4 weeks. Tumor volumes were measured regularly starting on the sixth day after implantation. The tumor growth was fit into an exponential growth curve (**Figure 4A**) (R^2^  = 0.87 and 0.84 for the DMH1 treated and control mice, respectively). The result indicated that the rate for doubling tumor size in DMH1-treated mice was about one day longer than the controls (5.6 versus 4.7 days in the DMH1 treated and control mice, respectively) (**Figure 4A**). As the initial tumor volumes were similar, no statistical differences between the two groups were observed until day 25. At the end of 4-week treatment, DMH1 treatment resulted in a statistically significant reduction in tumor volumes by about 50% compared to the vehicle control group (p-value <0.05) (**Figure 4B**). The mouse body weights were measured every other day throughout the experiment, and no notable weight changes were observed in both the control and DMH1 treated groups, suggesting an absence of DMH1 toxic effect at the administered dose (data not shown). To further examine the effect of DMH1 on tumor cell proliferation *in vivo*, tumor tissue samples from both the vehicle control and DMH1 treatment groups were subjected to Hematoxylin and eosin-stained (H&E) and human specific Ki67 staining. H&E sections were examined for regions that contained tumor and stromal cells, and the result indicated both the vehicle and DMH1 treated groups consisted of a morphologically similar differentiated adenocarcinoma (data not shown). However, immunohistochemical study showed a conspicuously significant decrease of human proliferation marker Ki67 in the DMH1 treated versus vehicle groups, suggesting that DMH1 treatment may attenuate human A549 cancer cell proliferation *in vivo* (**Figure 4C**).

## Discussion

BMPs are aberrantly expressed in many types of carcinoma cells including prostate, lung, breast, gastric and ovarian [16], [17], [18], [19]. For example, overexpression of BMP-2 is associated with ∼98% of NSCLC, but little or no activity of the BMP signaling cascade are detected in adult normal lung tissues, suggesting that blocking BMP signaling pathway may be an effective approach for lung cancer treatment [3], [4]. Indeed, the BMP antagonist Noggin and the extracellular pseudoreceptor spp24 were shown to reduce lung tumor growth in subcutaneous xenograft mouse models [6], [7]. Although the protein-based BMP antagonists and extracellular pseudoreceptors may be promising sources for lung cancer drug development, they have some potential limitations such as short half-lives and difficult delivery to the tumors [8]. In addition, both Noggin and spp24 block BMP signaling by interfering the binding of BMP ligands to their receptors at extracellular level, and their clinical application could be limited by any potential gain-of-function mutations in the downstream of the BMP signaling cascade. Small molecule antagonists like DMH1 that selectively target the intracellular kinase domains of BMP type I receptors would be ideal agents for the inhibition of lung cancer growth and metastasis. A recent study demonstrated that DMH2, one of BMP inhibitors identified in our zebrafish study effectively decreased growth and induced cell death of NSCLC cells *in vitro* [12]. Here we showed that a more selective BMP inhibitor, DMH1, significantly reduced NSCLC cell growth, migration and invasion *in vitro*, and attenuated tumor growth in the NSCLC xenograft mouse model, suggesting that inhibition of BMP pathway by antagonizing type I receptors with small molecules may be an effective approach for lung cancer therapy.

The effects of DMH1 on attenuating lung tumor growth may be mediated by multiple mechanisms, including disrupting the tumor microenvironment or lung cancer stem cells. Previous studies reported that BMP-2 induced the neovascularization of developing tumors, and stimulated blood vessel formation in A549-derived tumors in nude mice [20]. The BMP-2 antagonist noggin abrogated BMP-2-induced angiogenic response, and knocking down BMP-2 decreased blood vessel formation in the Matrigel assays [20]. Thus, DMH1 treatment may similarly disrupt microenvironment required for lung tumor growth by blocking angiogenesis. Additionally, BMP signaling is known in regulating stem cell self-renewal and differentiation, and some studies have suggested specific population of lung cancer cells display stem cell-like properties such as self-renewal and differentiation [21]. Blocking BMP signaling by DMH1 may interrupt lung cancer stem cell growth, thus suppressing tumor progression. This hypothesis is supported by a very recent study that inhibition of BMP signaling suppressed growth of the population of lung cancers cells expressing stem cell markers [22]. In addition, it has been reported from multiple studies that BMP-activated Smad signaling or the Id gene family have the potential to promote migration and invasion in different types of cancer [18], [23], [24]. Therefore, it is highly possible that the effect of targeting BMP signaling by DMH1 may reduce tumor invasion and metastasis in broader cancer categories.

In the in vivo study using the A549 xenograft model, the treatment was initiated on the same day of tumor cell implantation. Therefore, the tumor sizes were relatively small at the end of the in vivo experiments. In future studies, it remains to determine whether treatment by DMH1 following different schedules can result in greater anticancer effects. Although DMH1 treatment significantly attenuated lung cancer growth *in vivo*, compared with *in vivo* effects of chemotherapy drugs such as paclitaxel on the same xenograft model (unpublished results), the effect of DMH1 was not as potent. This observation suggests that DMH1 is not a cytotoxic agent and may not induce apoptosis at lower doses. Thus, for future clinical application, one of the potential research directions is to focus on the combined use of DMH1 with other targeted therapy or chemotherapy. A systematic approach to searching for a synergistic combination is needed. Further studies are also needed to identify subgroups of lung cancers that are more sensitive to BMP inhibitors for the purpose of individualized therapy.

In summary, our study demonstrated that antagonizing BMP type I receptors with small molecules is effective on suppressing lung cancer cell proliferation, migration, invasion *in vitro* and tumor growth *in vivo*. Non-toxic and highly selective small molecule BMP inhibitors, such as DMH1, may represent a novel class of therapeutic agents to reduce lung cancer patient's mortality and mobility.
